# Multicentre, adaptive, double-blind, three-arm, placebo-controlled, non-inferiority trial examining antimicrobial prophylaxis duration in cardiac surgery (CALIPSO): trial protocol

**DOI:** 10.1136/bmjopen-2025-115209

**Published:** 2026-03-18

**Authors:** Trisha Peel, David McGiffin, Julian Smith, Andrew Forbes, Silvana Marasco, David Pilcher, Andrew J Stewardson, Dennis Petrie, Anton Y Peleg, Jessica Wisniewski, Samuel Forster, Paige Druce, Janine Roney, Sarah Astbury, Danielle Berkovic, Phoebe Mccracken, Paul S Myles, on behalf of the CALIPSO Trial Steering Committee, Trisha Peel

**Affiliations:** 1 Infectious Diseases, Monash University, Melbourne, Victoria, Australia; 2 Infectious Diseases, Alfred Hospital, Melbourne, Victoria, Australia; 3 Cardiothoracic Surgery, Monash University Faculty of Medicine Nursing and Health Sciences, Melbourne, Victoria, Australia; 4 Alfred Hospital, Melbourne, Victoria, Australia; 5 Cardiothoracic Surgery, Monash Medical Centre Clayton, Clayton, Victoria, Australia; 6 Department of Surgery, School of Clinical Sciences, Monash University Faculty of Medicine Nursing and Health Sciences, Clayton, Victoria, Australia; 7 School of Public Health and Preventative Medicine, Monash University, Melbourne, Victoria, Australia; 8 Cardiothoracic Surgery, Alfred Hospital, Melbourne, Victoria, Australia; 9 Australian and New Zealand Intensive Care Society Centre for Outcomes and Resource Evaluation, Melbourne, Victoria, Australia; 10 Centre to Impact AMR, Monash University, Melbourne, Victoria, Australia; 11 Centre for Health Economics, Monash University, Melbourne, Victoria, Australia; 12 Department of Infectious Diseases, Monash University, Clayton, Victoria, Australia; 13 Infection Program, Monash University Monash Biomedicine Discovery Institute, Clayton, Victoria, Australia; 14 Hudson Institute of Medical Research, Clayton, Victoria, Australia; 15 Anaesthesiology and Perioperative Medicine, Monash University, Melbourne, Victoria, Australia; 16 Anaesthesiology and Perioperative Medicine, Alfred Health, Melbourne, Victoria, Australia; 17 Monash University, Clayton, Victoria, Australia; 18 Bayside Health, Melbourne, Victoria, Australia; 19 Monash University, Melbourne, Victoria, Australia; 20 Anaesthesiology and Perioperative Medicine, Alfred Hospital, Melbourne, Victoria, Australia

**Keywords:** SURGERY, Infection control, Antibiotics, Clinical Trial

## Abstract

**Introduction:**

Administration of antibiotics before incising the skin (‘surgical antimicrobial prophylaxis’) is a critical infection prevention strategy in surgery. Extending doses of prophylaxis into the postoperative period is a common practice in cardiac surgery; however, the benefit has not been clearly established and may lead to emergence of antimicrobial resistance and patient harm. We present the protocol for a large international multicentre, adaptive, pragmatic, double-blind, three-arm, placebo-controlled, randomised, non-inferiority clinical trial to compare the incidence of surgical site infection after three different durations of postoperative surgical antimicrobial prophylaxis in patients undergoing cardiac surgery.

**Methods and analysis:**

This adaptive, multi-arm multistage non-inferiority trial will compare intraoperative only (Arm A), to intraoperative and 24 hours (Arm B) and, to intraoperative and 48 hours (Arm C) of intravenous cefazolin and placebo as surgical antimicrobial prophylaxis in 9180 patients undergoing cardiac surgery. The adaptive design allows for potential dropping of any of the three arms if clear inferiority is indicated at any of the scheduled interim analyses. The trial will evaluate the clinical and cost-effectiveness of the three different antibiotic prophylaxis durations.

**Ethics and dissemination:**

Ethics approval will be obtained at all participating sites. Results of the study will be submitted for publication in peer-reviewed journals and the key findings presented at national and international conferences. Patients and members of the public will also be involved in the dissemination and translation of the trial results.

**Trial registration number:**

NCT05447559.

**STRENGTHS AND LIMITATIONS OF THIS STUDY**

- Comprehensive and adaptive trial design: This large, international, multicentre, double-blind, placebo-controlled, phase IV non-inferiority trial uses a robust adaptive framework with interim analyses and arm-dropping criteria, enhancing efficiency while maintaining statistical integrity and ethical oversight.
- Pragmatic approach enhances real-world relevance: By allowing perioperative care to reflect usual clinical practice, the study maximises external validity and applicability to routine cardiac surgical settings.
- Integrated economic and quality-of-life evaluation: The inclusion of health economic and quality of life analyses alongside clinical endpoints provides a multidimensional understanding of the impact of antimicrobial prophylaxis duration.
- Operational complexity across diverse settings: The global scale and adaptive design may introduce logistical challenges in maintaining protocol adherence, data quality and consistent blinding across multiple sites.

## Introduction

 Cardiac surgery is the highest volume major surgery performed in Australia^1^ with an estimated 1.5 million procedures performed globally each year.^2^ Postoperative infections following cardiac surgery have debilitating and life-threatening impacts on patients. Postoperative infections caused by surgical site infections (SSIs) and other healthcare-associated infections (HCAIs) complicate 8% and 15% of these operations, respectively.^3^,^5^ SSIs lead to patient suffering with mortality rates as high as 20%,^6^ and SSIs due to antimicrobial resistant bacteria are increasing,^7^ threatening to erode the potential life-saving benefits of this surgery.^8 9^ HCAIs due to *Clostridioides difficile* (*C. difficile*) are also increasing, directly linked to antimicrobial exposure, with mortality estimates of 12%.^10^,^12^ Together, the economic burden of these infections is substantial: data from the Alfred Hospital in Melbourne showed SSIs increase hospital costs by 150%^13^ while *C. difficile* infections in cardiac surgery patients in the USA cost the healthcare system ≥US$212 million each year.^11^

The risk of SSIs and other HCAIs can be reduced by administering antimicrobials (antibiotics) during the surgical procedure (‘intraoperative prophylaxis’).^14^ Current debate centres on whether there are net additional benefits with the administration of antimicrobials for variable durations after closure of the wound (‘postoperative prophylaxis’) in cardiac surgery.

The World Health Organisation (WHO)^15^ and US Centers for Disease Control and Prevention (CDC)^16^ released separate guidelines for SSI prevention and made recommendations against continuation of prophylaxis postoperatively for all surgery, including cardiac surgery. The guideline authors noted a potential benefit with extending prophylaxis to the postoperative period in cardiac surgery but based their decision against postoperative prophylaxis on the low quality of evidence and potential harms associated with prolonging antimicrobial therapy.^15 16^

Cardiac surgery has a unique set of circumstances that form the rationale for extending antimicrobial prophylaxis postoperatively, including use of cardiopulmonary bypass (associated with near-universal endotoxaemia/bacteraemia), hypothermia, prolonged operating times, intensive care unit (ICU) admission and mechanical ventilation postoperatively.^6 17 18^ Balanced against these arguments are the potential risks of additional antimicrobial exposure, such as the risk of *C. difficile*, alterations to the patient’s microbiome, acute kidney injury (AKI) and other patient harms.^10 19^ Data also suggest that longer durations of prophylaxis are associated with an increased risk of SSI, particularly due to antimicrobial resistant organisms.^20 21^ The WHO and CDC recommendations differ from US Society of Thoracic Surgeons guidelines and the Australian Therapeutic Guidelines, which recommend administration of prophylaxis postoperatively for ≤48 hours^6^ and ≤24 hours postoperatively, respectively.^22^ Thus, there is marked variations in policy and practice. With documented low adoption of these guidelines,^23 24^ the necessity of further clinical trial evidence is clear.

We identified three studies^5 25 26^ comparing intraoperative only to postoperative prophylaxis in cardiac surgery. The use of postoperative prophylaxis was associated with reduced odds of SSI (2.6% postoperative vs 5.8% intraoperative only: OR 0.44; 95% CI 0.26 to 0.72). Follow-up ranged from 7 days to 5 days, variable definitions of SSI were applied and, overall, the trials were of low quality.

We propose an adaptive, non-inferiority trial comparing intraoperative only (Arm A), to 24 hours (Arm B) and, to 48 hours (Arm C) of intravenously cefazolin and placebo postoperative surgical antimicrobial prophylaxis in 9180 patients undergoing cardiac surgery. Our trial will examine if:

1. Shorter prophylaxis durations are associated with a clinically acceptable low risk of SSI, specifically:
  - Intraoperatively only is non-inferior to intraoperatively plus 24 hours postoperatively.
  - Intraoperatively only is non-inferior to intraoperatively plus 48 hours postoperatively.
  - 24 hours postoperatively differs from 48 hours postoperatively.
2. The incidence of other HCAI, including *C. difficile*, is impacted by prophylaxis duration.
3. The relative cost-effectiveness of the different prophylaxis durations.

We will also conduct a substudy on the microbiological impact to determine whether longer durations of prophylaxis are associated with alterations to the normal microbiome and emergence of drug-resistant bacterial colonisation (protocol published separately). It is unlikely, from a biological perspective,^15 16 27^ that intraoperative (only) prophylaxis will be superior at preventing SSIs than postoperative doses. Intraoperative (only) prophylaxis, however, offers a potential safety advantage given reduced antimicrobial exposure may decrease *C. difficile* infections, reduce the drivers for antimicrobial resistance and also reduce other harms such as AKI. Intraoperative (only) prophylaxis may also reduce healthcare costs.^28^

### Stakeholder engagement and endorsement

The trial design is informed by feedback from end-users, data from real-world practice and, the discrepancies in guidelines including: (1) discordance in recommendations for duration of prophylaxis in cardiac surgery, ranging from intraoperative only,^15 16^ ≤24 hours postoperative^22 27^ and ≤48 hours postoperative^6^; (2) The Australian National Centre for Antimicrobial Stewardship and Australian Commission of Safety and Quality in Health Care National Antimicrobial Prescribing Survey^23 24^ data demonstrating heterogenous durations of prophylaxis in cardiac surgery; (3) stakeholder interviews have demonstrated lack of agreement and reluctance to change practice in the absence of a well-designed trial incorporating all current permutations of real-world practice.^23 24 29 30^

The CALIPSO Trial is endorsed by the Australian and New Zealand College of Anaesthetists Clinical Trials Network (ANZCA CTN), The Australian and New Zealand Intensive Care Society Clinical Trials Group and the Australasian Society for Infectious Diseases, and is supported by the Australian and New Zealand Society of Cardiac and Thoracic Surgeons. This multisite study will be coordinated by the ANZCA CTN and sponsored by Monash University, Melbourne, Australia. Clinical teams involved include infectious diseases, anaesthesia/perioperative medicine, cardiothoracic surgery and intensive care medicine.

## Methods and analysis

### Study design

This international, multicentre, adaptive, pragmatic, double-blind, three-arm, placebo-controlled, phase IV, non-inferiority trial will examine the incidence proportion of SSI following cardiac surgery. We will enrol 9180 patients from cardiac surgery centres throughout Australia and internationally (across New Zealand, Canada, Malaysia and the USA). Recruitment for this trial began in February 2023, with 2844 participants recruited as of January 2026 and will continue until December 2027. This is an effectiveness trial;^31 32^ therefore, some elements of the trial are deliberately left to the perioperative clinicians’ discretion in order to reflect usual practice and maximise generalisability. Our three-intervention trial will compare: administration of prophylaxis in the intraoperative period only (Arm A), administration of prophylaxis intraoperatively plus for 24 hours postoperatively (Arm B), and administration of prophylaxis intraoperatively plus for 48 hours postoperatively. The adaptive design allows for potential dropping of any of the three arms if clear inferiority is indicated at any of the scheduled interim analyses. The proposed trial will be conducted and reported in accordance with CONSORT (Consolidated Standards of Reporting Trials) and SPIRIT (Standard Protocol Items: Recommendations for Interventional Trials) guidelines.^33 34^

The primary endpoint for this trial is a composite endpoint comprised of the incidence of all SSI (superficial incisional SSI, deep and, organ/space SSI) at day 90 from index surgery (see online supplemental material). The definition is modified from the CDC National Healthcare Safety Network (NHSN) SSI surveillance definition.

The secondary endpoints of this trial are: the incidence of *C. difficile* infection at day 30 from index surgery; incidence of composite of all other HCAIs (pneumonia, blood stream infection and urinary tract infection) at index discharge, at death from any cause or at day 30 from index surgery—whichever came first, and incidence of late SSI occurring between day 91 and day 180; economic endpoints include: days alive and at home at 180 days (DAH_180_) after index surgery; direct healthcare costs in the 180 days after index surgery in those participants (Australian Sites and New Zealand Sites only); and quality of life measured by the five-level EuroQol five-dimensional questionnaire (EQ-5D-5L) at 30, 90 and 180 days after surgery (see online supplemental material for all endpoint definitions). The current protocol version is Number 6.0 (dated 9 October 2024).

### Non-inferiority margin

The US Food and Drug Administration and European Medicines Agency (EMA) have published guidance outlining approaches to determine the non-inferiority margin for non-inferiority trials.^31 35^ There are two approaches for determining the non-inferiority margin: the Fixed Margin Approach (denoted as M_1_) and the Synthesis Method (denoted as M_2_).

The Fixed Margin Approach (M_1_) estimates the active control effect based on analysis of past placebo-controlled trials. In this approach, the bound of the relevant 95% CI of the meta-analysis is typically selected as M_1_.^31 35^ The Synthesis Method (M_2_) is based on clinical judgement, that is, the largest clinically acceptable difference.

We undertook a systematic review and meta-analysis of randomised controlled trials comparing active antimicrobial prophylaxis to placebo in patients undergoing cardiac surgery. Four trials were identified.^32^,^3436^ The antimicrobials used in these trials were narrow-spectrum beta-lactams with similar activity and spectrum as cefazolin.^32^,^3436^ Comparing placebo to active antimicrobial prophylaxis, the pooled absolute risk difference for SSI was 15.8% (placebo 20.2% vs active prophylaxis 3.8%: 95% CI 8.1% to 23.5%).

This difference (8.1%) is higher than the anticipated effect size and is clinically unacceptable. We therefore adopted the Synthesis Method, deriving the largest clinically acceptable difference (M_2_) as 2.5% through agreement among clinical experts (also less than one-third of the difference of M_1_).

### Sample size

For this non-inferiority trial, there are three comparisons: (1) Arm A versus Arm B, (2) Arm A versus Arm C and (3) Arm B versus Arm C. Comparisons (1) and (2) are non-inferiority comparisons, and (3) is a superiority comparison (in either direction). The overall 5% significance level for the trial is partitioned into 1.67% for each of the three comparisons (1)–(3). The sample size to detect relevant differences (whether non-inferiority, harm or superiority) is based on numerical simulation of the primary outcome (SSI), details are below. With 9180 in each of the three equally sized groups of 3060 patients there is at least 80% power to detect a range of effects and allowing a 2% loss to follow-up.

The sample size calculation is based on:

1. A ‘base’ percentage of SSI of 8%, obtained from the incidence of SSI from prior randomised trials which recruited from the same population and applied the data collection methods and trial definitions and in keeping with the incidence reported by Tamayo *et al*.^3^,^5^
2. Non-inferiority limit (d) 2.5%.
3. The ‘stopping boundaries’ underlying the adaptive trial are based on:
  - Interim analyses are to be performed after 3060 and 6120 patients randomised, and
  - The interim analyses for each of three comparisons use symmetric O’Brien-Fleming boundaries with a two-tailed significance level of 1.67%, thereby controlling type I error rate at 5% across all comparisons at all time points.^37^ Should an arm be terminated at the first or second interim analysis, the remaining significance level will be reallocated to the comparison of the two continuing arms in the trial.

These boundaries, together with the consequent dropping of arms and reallocation of the remaining significance level to the two continuing arms, were simulated in 50 000 trials under a range of configurations to yield the following operating characteristics of the adaptive design:

- When the true SSI rates are 8% in each arm, the probability of correctly declaring non-inferiority (power) of the intraoperative arm compared with either of 24-hour and 48-hour arms is 88%, and power for simultaneous non-inferiority compared with both these arms is 80%.
- When the true SSI rates are 8% in the 24-hour and 48-hour arms and 10.5% in the intraoperative arm, representing the null configuration consisting of the threshold for inferiority (ie, harm) of the intraoperative arm and equality of the 24 and 48-hour arms, the probability of falsely declaring non-inferiority or harm of the intraoperative to either of the 24 or 48 hours arms was 1.66% and 1.61%, respectively, and the type I error rate for the comparison of 24 and 48 hours was 1.69%. These are all within sampling error of 1.67% and yield an overall familywise type I error rate of 4.39% across all three comparisons and three analysis time points.
- When the true SSI rates are 8% in the 24-hour and 48-hour arms and 13% in the intraoperative arm, representing clear harm, the probability of (correctly) declaring harm of the intraoperative arm (against either the 24-hour or 48-hour arms) is 89%.
  The type I error for the conventional comparison of 24 versus 48 hours is maintained at 1.67% in each of the above three configurations.
- When the SSI rates are 8% in the 24-hour and intraoperative arms, and (although unlikely) 13% in the 48-hour arm, the probability of dropping the 48-hour arm at an interim analysis is 99%, and the probability of (correctly) declaring non-inferiority of the intraoperative arm compared with 24 hours arm is 94%.

These results indicate that the adaptive design enables high power to detect non-inferiority when it truly exists and proper control of type I error rates, even when dropping inferior arms.

### Study population

Eligible patients are aged ≥18 years at the time of surgery and undergoing cardiac surgery involving a median sternotomy. Exclusion criteria are as follows:

- Age <18 years.
- American Society of Anesthesiologists physical status 5.
- Patients with a glomerular filtration rate (GFR) <40 mL/min/1.73 m^2^ or those requiring continuous renal replacement therapy, haemodialysis or peritoneal dialysis.
- Suspected or proven SSI at previous median sternotomy site.
- Patients currently receiving antibacterial (antibiotic) therapy preoperatively for treatment of a bacterial infection with a planned duration extending into the postoperative period.
- Documented cefazolin hypersensitivity or severe immediate or severe delayed hypersensitivity to beta-lactam antimicrobials.
- Surgery for suspected or proven endocarditis.
- Surgery for cardiac transplantation.
- Procedures involving insertion of a long-term (durable) ventricular assist device or mechanical circulatory support device.
- Procedures not involving a median sternotomy.
- Patients previously enrolled and randomised in the CALIPSO trial.

### Study intervention and blinding

**Figure 1:**
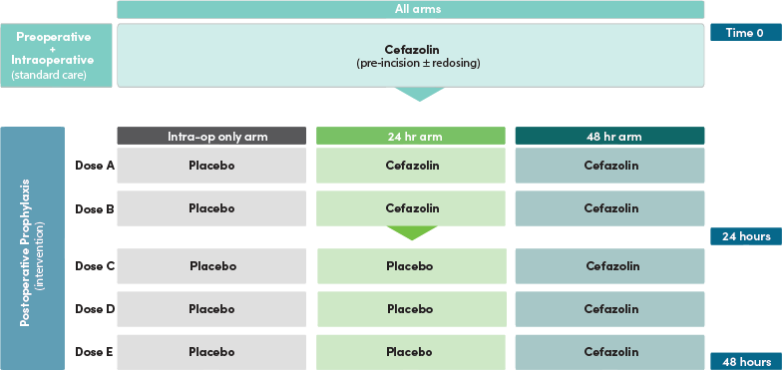
Trial intervention schema.

Following informed consent and eligibility screening, all participants will receive standard of care for intraoperative antimicrobial prophylaxis in accordance with the Australian Therapeutic Guidelines: Antibiotics,^22^ local or other national protocols or at the discretion of the treating clinician. The Australian Therapeutic Guidelines: Antibiotics recommends administration of intravenous cefazolin preoperatively within 60 min prior to skin incision (administration designated as ‘Time 0’), with intraoperative redosing every 3–4 hours if the procedure is prolonged.^22 27^ Our study examines the doses of prophylaxis administered following completion of the operation (ie, postoperative doses). The postoperative doses of study drug ideally will commence 8 hours from time 0 (ie, after the initial dose of standard of care antimicrobial prophylaxis administered at incision) and at the discretion of the treating clinician. Participants will be randomised to receive one of the three following intervention arms (figure 1):

Arm A: Placebo administered every 8 hours following the preoperative dose (time=0) for a total of five postoperative doses.

Arm B: Cefazolin administered 8-hourly following the preoperative dose (time=0) for two doses then placebo 8-hourly for three doses (total of five postoperative doses of cefazolin/placebo).

Arm C: Cefazolin administered every 8 hours following the preoperative dose (time=0) for a total of five postoperative doses.

Randomisation will be performed at the individual patient level. Participants will be randomly assigned in a ratio of 1:1:1 to the study arms in randomly sized blocks of a multiple of 3. Randomisation of participants to treatment arms will be as a password-protected, secure website using a central, computer-based randomisation programme with permutated blocks and allocation of participants stratified according to centre and diabetes status. The treating clinicians and study investigators will have no role in the assignment process. Participants, treating clinicians, all members of the research team including the study statistician will be blinded to treatment arm allocation. In the setting of a medical emergency, such as an adverse drug reaction, the code may be broken.

Active treatment will consist of 2 g cefazolin powder for injection. This dose is based on the Australian Therapeutic Guideline recommendations (2019)^22^ and is a pragmatic extension of current common practice. Placebo will consist of an identical empty vial. The vial containing cefazolin or placebo will be administered in keeping with product information for cefazolin and/or local guidelines. Cefazolin dissolves rapidly; however, there is a slight discolouration and odour with this drug. To ensure maintenance of blinding, the study drug will be drawn up and administered by the bedside nurse who will not be involved in the assessment and follow-up of the patient. The time and order of the vial administration (dose A – dose E) will be documented. With the exception of the bedside nurse administering and/or drawing up the study drug, all clinicians and research team members will be blinded to arm allocation. At some international sites, local supplies of generic (cefazolin) drug or placebo may be used. At international sites preparing the study drug, cefazolin 2 g will be prepared by a pharmacist in an International Organization for Standardization (ISO) 5 environment and stored in keeping with the product information and/or local pharmacy operating procedures. The placebo will be prepared by the pharmacy department to match the reconstituting fluid type and volume of the active study product. For example, if the cefazolin is diluted in 100 mL of sodium chloride 0.9%, the placebo will consist of the sodium chloride 0.9% made up to equal the volume of the active study drug. Members of the pharmacy team preparing the study drug will not be involved in the operation or trial data collection. The study drug doses will be labelled with the sequential order of administration (dose A – dose E) in a carton (kit) or individual bags. With the exception of the pharmacy department preparing the study drug, all clinicians and research team members will be blinded to arm allocation.

The postoperative administration of other antimicrobials for the purpose of postoperative surgical antimicrobial prophylaxis (eg, administration of postoperative doses of vancomycin for surgical antimicrobial prophylaxis) will not be allowed. Antimicrobials may be administered in the postoperative period for the prevention of other infections in accordance with local guidelines/practice (such as urinary tract infection in the setting of urinary catheters) or for the treatment of postoperative infections (such as pneumonia) at the direction of the treating clinician.

### Data collection and outcome methods

**Figure 2:**
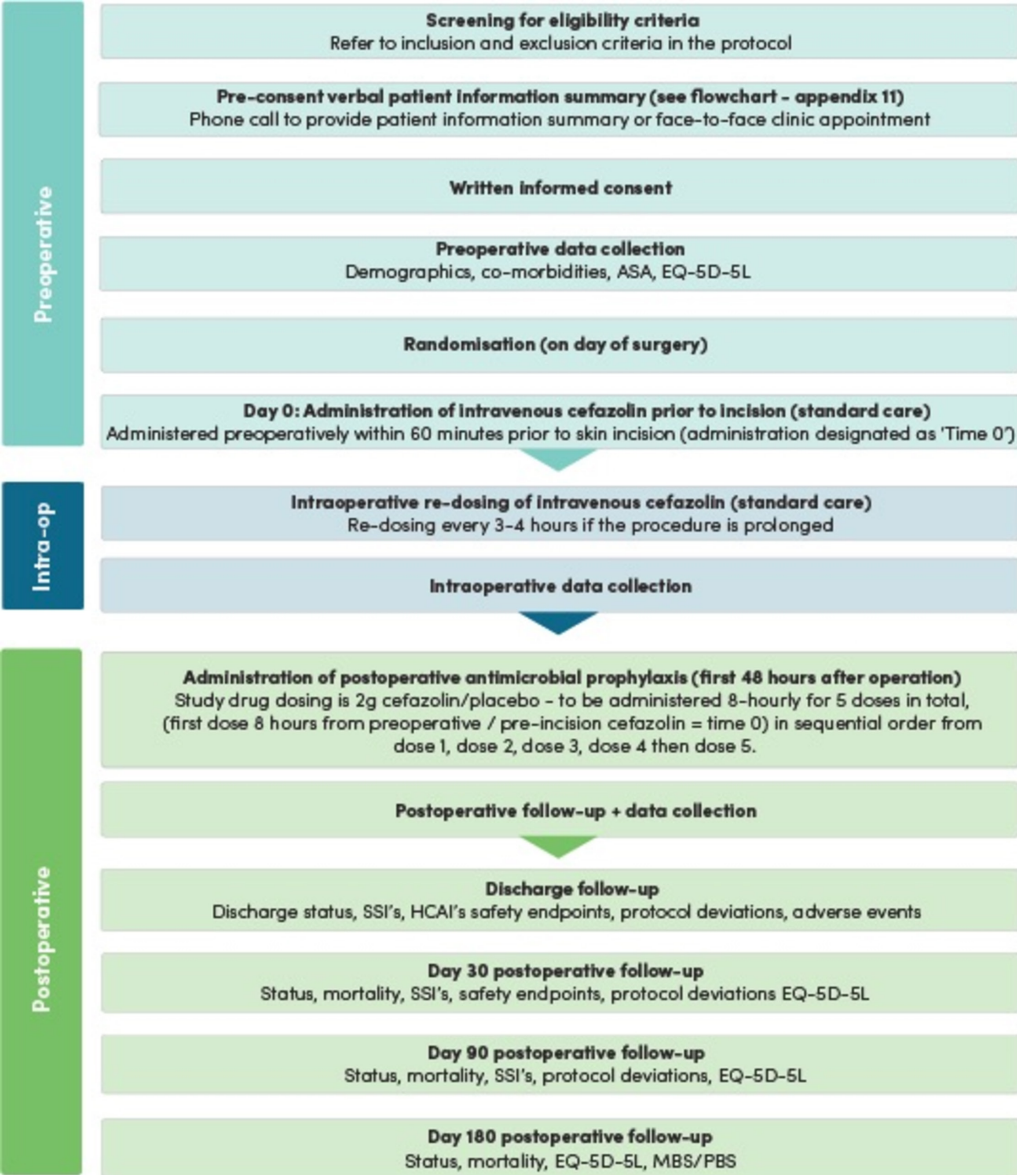
Trial schema. ASA, American Society of Anesthesiologists; EQ-5D-5L, five-level EuroQol five-dimensional questionnaire; HCAIs, healthcare associated infections; MBS, Medicare Benefits Schedule; PBS, Pharmaceutical Benefits Scheme; SSIs, surgical site infections.

Participants will be followed for 180 days from index surgery to capture all relevant clinical and health economic outcomes (figure 2). Demographic data, results from serum urea, electrolytes and creatinine, full blood examination and other laboratory testing, operative data and quality of life (EQ-5D-5L) will be collected. Information on all aspects of intraoperative antimicrobial prophylaxis will be collected, including re-dosing (time, dose and method of administration), as well as operation time, and commencement, cessation (and duration) of bypass. Information regarding other infection prevention approaches (including screening and decolonisation, and surgical site skin preparation) will also be collected and reported descriptively to assess balance between arms.

On the day of discharge, participants will be assessed for occurrence of infection including SSI (primary endpoint) or HCAIs (secondary endpoint), adverse events and results of laboratory investigations. Data will be collected on any wound complications since prior review at day 30, 90 and 180. The occurrence of *C. difficile* infection since prior review will be assessed at day 30 only.

### Endpoint adjudication

Participants will be followed for 90 days for clinical endpoints and 180 days for economic outcomes. At each contact, the participant will be questioned as to the occurrence of the study outcome of interest in the period since last contact. Notification of a potential study outcome will trigger the collection of information for review by the Endpoint Verification Coordinator and Endpoint Adjudication Committee.

The processes for identifying outcomes will be conducted by the project research officer, who will collect demographic data and relevant laboratory testing, operative data and quality of life (EQ-5D-5L). Active surveillance will comprise the following:

- Review of all participants’ medical records on discharge and follow-up.
- Telephone contact for outcomes and quality of life data at 30, 90 and 180 days following index surgery.
- Contact with the participant’s general practitioner and other healthcare institutions as required.
- Clinical microbiological reports with antimicrobial susceptibility data for SSI and HCAI isolates.

In participants meeting the criteria for the primary outcome, secondary outcomes of interest or adverse events outcomes, de-identified data will be forwarded to the CALIPSO Clinical Trial Coordinating Centre, Department of Infectious Diseases, Monash University, for endpoint adjudication. De-identified data will include:

- Details from the participant’s medical records.
- Medical records obtained from other healthcare institutions.
- Hospital records/discharge summaries and pathology report.
- Relevant microbiology reports.

Ascertainment of outcomes will be undertaken according to review against strict criteria. Endpoints meeting the definitions will be recorded as such. There will be a two-step endpoint verification/adjudication process. A custom algorithm is built into the database and endpoint submission form, to ensure that reports of endpoints either comply with CDC criteria, or where deficient, to prompt a request for further information. The de-identified endpoint data will first be sent to the Endpoint Verification Coordinator. If further adjudication is required to determine the outcome of the endpoint, the information will then be forwarded to the Endpoint Adjudication Committee. This committee consists of the Chief Principal Investigator (CPI) and two experienced infectious diseases physicians. The CPI and one endpoint committee member will review the information. If a consensus cannot be attained, the information will be sent to the third adjudicator, so that a majority decision may be reached.

### Trial outcomes

Primary: The primary endpoint for this trial is a composite endpoint comprised of the incidence of all SSI (superficial incisional SSI, deep and, organ/space SSI) at day 90 from index surgery (see online supplemental material). The definition is modified from the CDC NHSN SSI surveillance definition. Superficial, deep and organ/space SSIs will be reported separately as secondary or exploratory analyses, in addition to the composite primary endpoint.

Secondary: The secondary endpoints of this trial are (for definitions see online supplemental material):

- Incidence of *C. difficile* infection at day 30 from index surgery.
- Incidence of composite of all other HCAIs (pneumonia, blood stream infection and urinary tract infection) at index discharge, at death from any cause or at day 30 from index surgery—whichever came first.
- Incidence of late SSI occurring between day 91 and day 180.

Economic endpoints (see definitions online supplemental material) include:

- Days alive and at home at 180 days (DAH_180_) after index surgery.
- Direct healthcare costs within 180 days after index surgery (Australian Sites and New Zealand Sites only).
- Quality of life (EQ-5D-5L) measured at 30, 90 and 180 days after surgery.

Safety endpoints are the incidence of adverse events (see definitions in online supplemental material) including:

- Antimicrobial hypersensitivity reactions at index discharge, at death from any cause or at day 30 from index surgery—whichever came first.
- All-cause mortality at 180 days from index surgery.
- SSIs due to drug-resistant infections (defined as resistance to cefazolin) at day 90 from index surgery.
- Acute kidney injury at index discharge, at death from any cause or at day 30 from index surgery—whichever came first.

### Statistical analysis

The analysis and reporting of the results will follow the CONSORT guidelines.^33^ Data will be summarised with respect to baseline characteristics, primary and secondary endpoints and safety endpoints. Data will be presented in summary tables according to treatment allocation. As a large randomised controlled trial, covariate balance is expected and no covariate adjustment will be performed apart from adjustment for the randomisation stratification factor of diabetes status.

We will employ a modified intention-to-treat (mITT) principle for the primary outcome in the mITT population of all randomised participants who undergo eligible cardiac surgery, corresponding to a treatment policy estimand. We will also perform a per-protocol analysis which includes participants who completed the treatment to which they were allocated, meaning only those who receive all doses of cefazolin/placebo according to their original allocation, which corresponds to a hypothetical estimand of full compliance. Both estimand strategies are explicitly recognised in (and are compliant with) the International Council for Harmonisation of Technical Requirements for Pharmaceuticals for Human Use (ICH) E9(R1) Addendum. Analysis of the primary outcome will report a two-sided CI, adjusted for multiplicity of interim analyses assessments using binomial regression with an identity link function to estimate the difference in SSI rates directly, adjusted for diabetes status at randomisation. Per-protocol analyses will be performed similarly, but in addition using inverse probability weighting to account for potential selection bias. If both approaches support non-inferiority, the comparator arm will be considered not inferior. Additional analyses will be carried out on prespecified subgroups (age strata, sex, body mass index). The same statistical model will be used for binary secondary and safety outcomes, and with continuous outcomes analysed using linear or quantile regression as appropriate depending on their skewness. Missing outcome data will be multiply imputed if the rate of missingness exceeds 5%.

**Figure 3:**
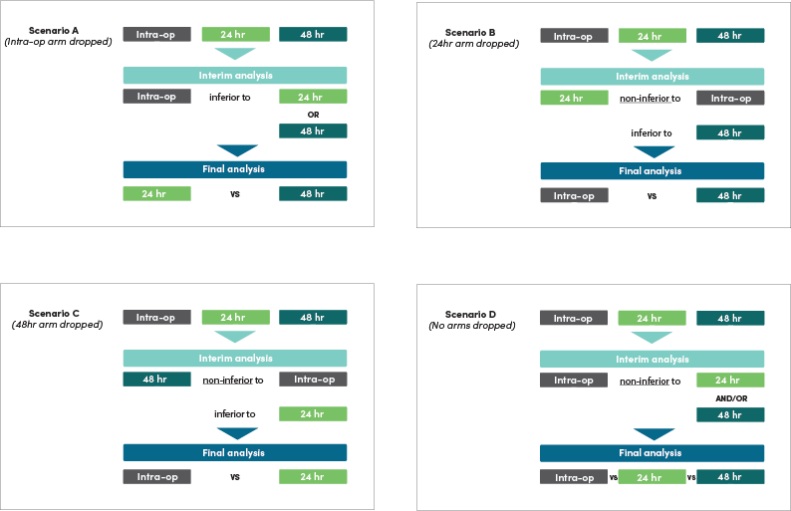
Interim analysis scenarios.

The adaptive design allows the potential dropping of any of the three arms if clear inferiority is indicated at any of the scheduled interim analyses. These analyses will be limited to consideration of the primary outcome (incidence of all SSI). Dropping of the intraoperative only arm (Arm A) may be considered if an interim analysis demonstrates that Arm A is inferior to either 24 hours (Arm B) or 48 hours (Arm C) postoperative prophylaxis (Scenario A: figure 3). Such inferiority would require the lower endpoint of the relevant confidence interval (adjusted for interim analyses) for the difference in SSI rates (intraoperative minus 24 or 48 hours) to lie above the non-inferiority margin of 2.5% The trial would then continue randomising to 24 (Arm B) or 48 hours (Arm C) to provide the definitive answer. Similarly, if either the 24 hours (Arm B) or 48 hours (Arm C) demonstrates conclusive evidence of a true difference in their head-to-head comparison at interim analysis (Scenario B and C, respectively, figure 3), via the CI for the difference excluding the zero point, dropping the inferior of the two arms may be considered, and the trial continued with just the superior arm and the intraoperative arm (subject to it too not demonstrating inferiority). If the interim analyses show intraoperative only (Arm A) is non-inferior to one or both of 24 (Arm B) and 48 hours (Arm C), as evidenced by the upper endpoint of the CI for the difference in SSI rates lying below 2.5% (Scenario D: figure 3), the trial will not terminate, as there is no ethical dilemma in continuing, and given the importance of determining with maximum certainty the optimal duration of prophylaxis to assist with translation of these currently embedded practices.^38 39^ Early stopping of any arm may also be considered by the Data and Safety Monitoring Committee (DSMC) based on designated safety endpoints. Stopping the trial for futility will not be performed.

The economic evaluation will take a healthcare perspective to estimate the cost per SSI and/or other HCAI avoided and the cost per quality adjusted life year (QALY) gained between the three arms. The cost differences will include the cost savings of not having to administer postoperative antimicrobial prophylaxis in Arms B and C, respectively, along with the downstream cost implications associated with differences in infections, complications and length of hospital stay (up until 180 days) across the three arms. The downstream costs will be based on administrative data on the resources used from the hospital sites (Australian and New Zealand sites only) with unit costs obtained from the literature. The difference in SSI and HCAI between protocol arms will be based on the primary analysis.

In terms of QALYs—those who die within the first 30 days will be assumed to have zero QALYs regardless of the days they survive. QALYs within the first 30 days post the index surgery will be based on a zero QALY weight for any ICU stay, and a linear improvement from zero on the date of surgery or last ICU stay to their reported 30-day QALY weight. QALY weights from 30 days to 180 days will be based on linear extrapolation between QALY weight at each follow-up point or to zero when an individual is recorded to have died. First, a complete case analysis will be performed and the robustness to missing data explored using multiple imputation. A probabilistic sensitivity analysis will be undertaken to explore the robustness of the results to the uncertainty around the parameters.

The complete analysis plan will be finalised prior to the conduct of the first planned interim analysis (when 3060 participants have been randomised) and will be made publicly available.

### Planned substudies

There are two planned substudies for the trial, the protocols for which will be published separately:

- Genomics substudy: conducted at Alfred Hospital and will examine whether differing antimicrobial prophylaxis durations:
  - Impact on the microbial diversity of the gut.
  - Drive the emergence of antimicrobial resistance.
  - Lead to sustained changes to diversity or antimicrobial resistance.
  - Impact on host inflammatory-immune response.
- Postoperative infection pathogen substudy: this substudy will include the collection of pathogens isolated from clinical specimens at Alfred Hospital.

### Patient and public involvement

Patients and members of the public have been involved at several stages of the trial, including the design, management and conduct of the trial. The specific choice of intervention arms and trial endpoints reflects the feedback from stakeholders and consumers in the conceptualisation and designs phase. The CALIPSO steering and operations committees engage with consumers at the partnership level, with a consumer representative member on the trial steering committee to facilitate ongoing feedback regarding the management and conduct of the trial. A consumer framework has been developed to ensure consumer engagement has been formalised, and consumers receive adequate training and remuneration for their time. Consumers will also be involved in the dissemination and translation phase of the trial.

## Ethics and dissemination

This study will be conducted in accordance with the ICH GCP notes for Guidance on Good Clinical Practice (CPMP/ICH/135/95) and as defined by the International Conference on Harmonisation. As of January 2026, the trial has received approval from The Alfred Hospital Ethics Committee (local reference: 86287), Melbourne, Australia (under the National Mutual Acceptance scheme), as well as by St John of God Health Care Human Research Ethics Committee (local reference: 2037) Australia, UnitingCare Health Human Research Ethics Committee (local reference: 202412) Australia, the Central Health and Disability Ethics Committee (NZ HDEC) New Zealand (local reference: 2023 FULL 15380), the Nova Scotia Health Research Ethics Board Canada (local reference: REB File #:1031513), the University of Texas Southwestern Institutional Review Board USA (local reference: STU-2024-0764) and the Institut Jantung Negara Research Ethics Committee (IJNREC) Malaysia (local reference: IJNREC/740/2025). The protocol for this study will be submitted to all involved universities and participating hospitals for assessment and approval. The conduct of this study will conform to the Australian Health Ethics Committee (AHEC) guidelines for human research. Participant recruitment will not commence until approval by the properly constituted and accredited Human Research Ethics Committee or institutional review board and the relevant site regulatory body has been obtained.

Informed consent will be obtained for all participants. The investigator will retain each patient’s signed consent form (if written form), or in those participants that provide verbal consent, the designated study personnel will retain the record of the participant’s response in writing or on an audio device. Patients will be advised that they are free to refuse to participate in, or to withdraw from the study at any time. The medical care provided will not be affected by agreement or refusal to participate in this study.

All case report forms (CRFs) and all other documents associated with this study must be archived for at least 15 years following the completion of the trial, in accordance with Therapeutic Goods Association (TGA) requirements, or in accordance with the regulatory and legal requirements as specified in each region. Full identification of each participant will be kept by the local site investigator/research staff. All information will be treated in accordance with professional conduct. Confidentiality of all participant information will be maintained and stored in accordance with policy.

### Safety

An independent DSMC will review the interim analyses, monitor data and safety endpoints, data from external sources and evaluate study progress.

Cefazolin is an internationally approved antimicrobial and is indicated for surgical antimicrobial prophylaxis as per the Australian Therapeutic Guidelines: Antibiotics.^22^ Adverse reactions such as AKI and hypersensitivity reactions are well-characterised and reported associations of this therapy. These adverse outcomes will be collected and analysed as part of the trial protocol. The trial drug will be given in the postoperative period for five doses postoperatively, ceasing 48 hours from the initial preoperative dose (time 0). Reportable side effects from drug administration will occur during administration or within 30 days of the infusion.

The safety endpoints for this trial are: antimicrobial hypersensitivity reactions at index discharge, at death from any cause or at day 30 from index surgery—whichever came first; all-cause mortality at 180 days from index surgery; surgical site infections due to drug-resistant infections (defined as resistance to cefazolin) at day 90 from index surgery; AKI at index discharge, at death from any cause or at day 30 from index surgery—whichever came first (see online supplemental material for full definitions). The CRF has been designed to capture safety endpoints following surgery routinely, such as admission to the ICU, need for mechanical ventilation, AKI and postoperative infections, to enable the DSMC to review data in a meaningful way, by independent experts, with blinded separation of treatment groups enabling useful comparisons for the whole dataset.

In the event of a hypersensitivity reaction, it will be assumed that the participant received cefazolin prophylaxis and administration of the prophylaxis will be ceased. In the event of a clinical emergency, such as an adverse drug reaction, the code can be broken by contacting the Monash Department of Infectious Diseases Research Office to allow emergency un-blinding.

Adverse events (including serious adverse events, and suspected unexpected serious adverse reactions) will be captured and reported in accordance with ICH GCP notes for Guidance on Good Clinical Practice (CPMP/ICH/135/95) and as defined by the International Conference on Harmonisation and will be identified by system and severity. Sites will be required to report any adverse events that occur during the perioperative period.

A Data Quality Committee will monitor and report on data accuracy and completeness for the CALIPSO trial. For the purpose of compliance with ICH GCP notes for Guidance on Good Clinical Practice (CPMP/ICH/135/95), random audits of centres will be undertaken to assess the accuracy and legitimacy of the trial data. Statistical monitoring of the data completeness, data variance and risk-appropriate endpoint rates will be done for all patient data.

### Dissemination

The results of this trial will be submitted for publication in peer-reviewed journals and the key findings presented at national and international conferences. Results will also be disseminated to key professional groups, and regulatory and funding bodies.

All planned publications arising from this project will be submitted to the steering committee for consideration and authorisation. Criteria for authorship on any publications will be in keeping with the Australian National Health and Medical Research Council^40^ guide for authorship and the International Committee of Medical Journal Editors criteria.^41^

## Supplementary material

10.1136/bmjopen-2025-115209 online supplemental file 1
