# Supplementary material for: Multicentre, adaptive, double-blind, three-arm, placebo-controlled, non-inferiority trial examining antimicrobial prophylaxis duration in cardiac surgery (CALIPSO): trial protocol
Source: BMJ Open. 2026 Mar 18;16(3):e115209. doi: 10.1136/bmjopen-2025-115209 (PMC13007059; doi:10.1136/bmjopen-2025-115209)
Supplement: online supplemental file 1 [file bmjopen-16-3-s001.docx]

**SUPPLEMENTAL MATERIAL**

# **Primary Endpoint Definitions**

Primary endpoints are modified from the Centers for Disease Control (CDC)/National Healthcare Safety Network (NHSN) surveillance definitions for surgical site infections (SSI) following cardiac surgery.

| **ENDPOINT** | **DEFINITIONS** |
| --- | --- |
| Superficial Incisional SSI | Must meet the following criteria:   1. Date of event occurs within 90 days after index cardiac surgery   (where day 1 = the procedure date)  AND   1. Involves only skin and subcutaneous tissue of the incision*   AND   1. Patient has **at least one** of the following:    1. purulent drainage from the superficial incision.    2. organism(s) identified from an aseptically-obtained specimen from the superficial incision or subcutaneous tissue by a culture    3. superficial incision that is deliberately opened by a clinician ^Ψ^ and culture of the superficial incision or subcutaneous tissue is not performed   AND  patient has at least one of the following signs or symptoms: localized pain or tenderness; localized swelling; erythema; or heat.   - 1. diagnosis of a superficial incisional SSI by a clinician ^Ψ^   Notes:  * For CALIPSO, there are two specific types of superficial incisional SSIs:   - Superficial Incisional Primary (SIP) – a superficial incisional SSI that is identified in the primary incision in a patient that has had an operation with one or more incisions (for example, chest incision for median sternotomy) - Superficial Incisional Secondary (SIS) – a superficial incisional SSI that is identified in the secondary incision in a patient that has had an operation with more than one incision (for example, donor site incision for coronary artery bypass graft)   ^Ψ^ The term clinician for the purpose of application of the CALIPSO endpoint criteria may be interpreted to mean a surgeon, infectious disease physician, emergency physician, other physician on the case, or local medical officer/primary care physician/general practitioner, or nurse practitioner or infection prevention nurse. |
| Deep incisional SSI | Must meet the following criteria:   1. The date of event occurs within 90 days after index cardiac surgery   (where day 1 = the procedure date)  AND   1. involves deep soft tissues of the incision* (for example, fascial and muscle layers)   AND   1. patient has **at least one** of the following:    1. purulent drainage from the deep incision.    2. a deep incision that spontaneously dehisces, or is deliberately opened or aspirated by a clinician ^Ψ^   AND  organism(s) identified from the deep soft tissues of the incision OR microbiologic testing is not performed.^†^  AND  patient has at **least one** of the following signs or symptoms: fever (>38°C); localized pain or tenderness.   - 1. an abscess or other evidence of infection involving the deep incision that is detected on gross anatomical or histopathologic exam, or imaging test.   Notes:  * For CALISPO, there are two specific types of deep incisional SSIs:   - Deep Incisional Primary (DIP) – a deep incisional SSI that is identified in the primary incision in a patient that has had an operation with one or more incisions (for example, chest incision for median sternotomy) - Deep Incisional Secondary (DIS) – a deep incisional SSI that is identified in the secondary incision in a patient that has had an operation with more than one incision (for example, donor site incision for coronary artery bypass graft)   ^Ψ^ The term clinician for the purpose of application of the CALIPSO endpoint criteria may be interpreted to mean a surgeon, infectious disease physician, emergency physician, other physician on the case, or local medical officer/primary care physician/general practitioner, or nurse practitioner or infection prevention nurse.  ^†^ Culture from the deep soft tissues of the incision that has a negative finding does not meet this criterion |
| Organ/Space SSI | Must meet the following criteria:   1. The date of event occurs within 90 days after index cardiac surgery   (where day 1 = the procedure date)  AND   1. involves any part of the body deeper than the fascial/muscle layers that is opened or manipulated during the operative procedure   AND   1. patient has **at least one** of the following:    1. purulent drainage from a drain that is placed into the organ/space (for example, closed suction drainage system, open drain, CT-guided drainage).    2. organism(s) identified from fluid or tissue in the organ/space    3. an abscess or other evidence of infection involving the organ/space that is detected on gross anatomical or histopathologic exam, or imaging test evidence suggestive of infection.   AND   1. meets **at least one** criterion for a specific organ/space infection site (defined below):    1. Myocarditis or pericarditis    2. Endocarditis    3. Mediastinitis    4. Sternal Osteomyelitis |
| Myocarditis or pericarditis | Must meet the definition for Organ/Space SSI  AND  Must meet **at least one** of the following criteria:   1. Patient has organism(s) identified from pericardial tissue or fluid 2. Patient has **at least two** of the following signs or symptoms:   fever (>38.0°C), chest pain*, paradoxical pulse*, or increased heart size*  AND **at least one** of the following:   - 1. ECG consistent with myocarditis or pericarditis   2. evidence of myocarditis or pericarditis on histologic exam of heart tissue   3. pericardial effusion identified by echocardiogram, CT scan, MRI, or angiography.   * With no other recognized cause |
| Endocarditis | Must meet the definition for Organ/Space SSI  AND  Must meet **at least one** of the following criteria:   - - - 1. Organism(s) identified from cardiac vegetation*†, embolized vegetation (for example, solid-organ abscess) documented as originating from cardiac source, or intracardiac abscess       2. Organism(s) seen on histopathologic examination of cardiac vegetation*, embolized vegetation, for example, solid organ abscess, documented as originating from cardiac source, or intracardiac abscess.   OR   - - - 1. Endocarditis seen on histopathologic examination of cardiac vegetation* or intracardiac abscess.       2. At least one of the following echocardiographic evidence of endocarditis*‡:   1. vegetation on cardiac valve or supporting structures   2. intracardiac abscess   3. new partial dehiscence of prosthetic valve   AND **one** of the following   - - 1. Typical organism(s)^^ isolated from ≥2 blood collections drawn on separate occasions     2. *Coxiella burnetii* identified by anti-phase I IgG antibody titer >1:800 or non-culture based microbiologic testing method (eg. 16sRNA)        1. At least **three** of the following:           1. Prior endocarditis, prosthetic valve, uncorrected congenital heart disease, history of rheumatic heart disease, hypertrophic obstructive cardiomyopathy           2. Fever (>38.0°C)           3. Vascular phenomena^⌃^           4. Immunologic phenomena^♦^           5. AND **one** of the following   Typical organism(s)^♤^ isolated from ≥2 blood collections drawn on separate occasions  *Coxiella burnetii* identified by anti-phase I IgG antibody titer >1:800 or non-culture based microbiologic testing method (eg. 16sRNA)   - - - 1. At least **one** of the following*‡:     1. Vegetation on cardiac valve or supporting structures seen on echocardiogram     2. Intracardiac abscess seen on echocardiogram     3. New partial dehiscence of prosthetic valve seen on echocardiogram     4. AND at least **three** of the following  1. prior endocarditis, prosthetic valve, uncorrected congenital heart disease, history of rheumatic heart disease, hypertrophic obstructive cardiomyopathy. 2. fever (>38.0°C) 3. Vascular phenomena^⌃^ 4. Immunologic phenomena^♦^ 5. Identification of organism(s) from the blood by at least one of the following methods:    - Typical organism^♤^ identified from blood culture    - Commensal organism(s)^♣^ identified from ≥2 blood cultures drawn on separate occasions on the same or consecutive days   7. **All** of the following criteria:   - 1. Prior endocarditis, prosthetic valve, uncorrected congenital heart disease, history of rheumatic heart disease, hypertrophic obstructive cardiomyopathy.  1. Fever (>38.0°C) 2. Vascular phenomena^⌃^ 3. Immunologic phenomena^♦^ 4. Identification of organism(s) from the blood by at least one of the following methods:  - Typical organism^♤^ identified from blood culture - Commensal organism(s)^♣^ identified from ≥2 blood cultures drawn on separate occasions.   Notes  * Cardiac vegetation can be found on a cardiac valve, pacemaker/defibrillator lead or ventricular assist device (VAD) components within the heart.  † The following can also meet the definition of a “cardiac vegetation”: Positive culture from a cardiac valve, pacemaker/defibrillator lead or ventricular assist device (VAD) components within the heart.  ‡ Which if equivocal is supported by clinical correlation (specifically, physician documentation of antimicrobial treatment for endocarditis).  ^♤^ Typical organisms include Viridans group streptococci, Streptococcus bovis, Haemophilus spp., Actinobacillus actinomycetemcomitans, Cardiobacterium hominis, Eikenella corrodens, Kingella spp., Staphylococcus aureus, Enterococcus spp.  ^⌃^ Vascular phenomena include: major arterial emboli (specifically, embolic stroke, renal infarct, splenic infarct or abscess, digital ischemic/gangrene from embolic source), septic pulmonary infarcts, mycotic aneurysm (documented by imaging, seen in surgery, or described in gross pathological specimen), intracranial hemorrhage, conjunctival hemorrhages, or Janeway’s lesions documented  ^♦^ Immunologic phenomena include: glomuleronephritis (documented in chart, or white cell or red blood cell casts on urinalysis), Osler’s nodes, Roth’s spots, or positive rheumatoid factor  ^♣^ Commensal organisms include: diphtheroids (Corynebacterium spp. not C. diphtheria), Bacillus spp. (not B. anthracis), Propionibacterium spp., coagulase-negative staphylococci (including S. epidermidis), viridans group streptococci, Aerococcus spp. Micrococcus spp. and Rhodococcus spp. |
| Mediastinitis | Must meet the definition for Organ/Space SSI  AND  Must meet **at least one** of the following criteria:   - - - 1. Patient has organism(s) identified from mediastinal tissue or fluid       2. Patient has evidence of mediastinitis on gross anatomic or histopathologic exam.       3. Patient has **at least one** of the following signs or symptoms: fever (>38.0°C), chest pain*, or sternal instability*   AND **at least one** of the following:   - - - - 1. purulent drainage from mediastinal area         2. mediastinal widening on imaging test   * with no other recognized cause  Comments  The mediastinal space is the area under the sternum and in front of the vertebral column, containing the heart and its large vessels, trachea, oesophagus, thymus, lymph nodes, and other structures and tissues. It is divided into anterior, middle, posterior, and superior regions. |
| Sternal osteomyelitis | Must meet the definition for Organ/Space SSI  AND  Must meet **at least one** of the following criteria:   1. Patient has organism(s) identified from sternal bone culture 2. Patient has evidence of sternal osteomyelitis on gross anatomic or histopathologic exam. 3. Patient has **at least two** of the following localized signs or symptoms: fever (>38.0°C), swelling*, pain or tenderness*, heat*, or drainage*   And **at least one** of the following:   - 1. organism(s) identified from blood culture   AND  imaging test evidence suggestive of infection (for example, x-ray, CT scan, MRI, radiolabel scan [gallium, technetium, etc.]), which if equivocal is supported by clinical correlation, specifically, physician documentation of antimicrobial treatment for osteomyelitis.   - 1. imaging test evidence suggestive of infection (for example, x-ray, CT scan, MRI, radiolabel scan [gallium, technetium, etc.]), which if equivocal is supported by clinical correlation, specifically, physician documentation of antimicrobial treatment for osteomyelitis.   * With no other recognized cause |

**2.** **Secondary Endpoint Definitions**

Secondary endpoints are based on the CDC/NHSN definitions for Healthcare Associated Infections (HCAI).

| **ENDPOINT** | **DEFINITIONS** |
| --- | --- |
| *Clostridioides difficile* infection [CDI]^67^ | For the purposes of CALIPSO study, this infection must occur within 30 days after index cardiac surgery (where day 1 = the procedure date).  AND  Was not found to be present or incubating at the time of index admission  AND  Must meet **at least one** of the following criteria:   1. Positive test for toxin-producing *C. difficile* on an unformed stool specimen (conforms to the shape of the container). 2. Patient has evidence of pseudomembranous colitis on gross anatomic (includes endoscopic exams) or histopathologic exam.   Comments:  • The date of event for CDI criterion 1, will always be the specimen collection date of the unformed stool, specifically, not the date of onset of unformed stool.  • A positive test for toxin-producing C. difficile and an unformed stool specimen is a single element, and both are required to meet criterion |
| Blood Stream Infection [BSI] ^69^ | For the purposes of CALIPSO study, this infection must occur within index acute healthcare admission after index cardiac surgery (excludes admission to rehabilitation or sub-acute care facilities).Time frame of measurement for this endpoint will be from date of index surgery until the date of discharge from the acute healthcare facility or date of death from any cause, whichever came first, assessed up to 30 days from index surgery.  AND  Was not found to be present or incubating at the time of index admission  AND  Must meet the following criteria:   1. Patient has a recognized bacterial or fungal pathogen, not included on the NHSN common commensal list, identified from one or more blood specimens obtained by a culture   AND  Organism(s) identified in blood is not related to an infection at another site  OR   1. Patient has at least one of the following signs or symptoms: fever (>38.0^o^C), chills, or hypotension   AND  Organism(s) identified in blood is not related to an infection at another site  AND  The same NHSN common commensal is identified by a culture from two or more blood specimens collected on separate occasions. Common Commensal organisms include, but are not limited to, diphtheroids (Corynebacterium spp. not C. diphtheria), Bacillus spp. (not B. anthracis), Propionibacterium spp., coagulase-negative staphylococci (including S. epidermidis), viridans group streptococci, Aerococcus spp. Micrococcus spp. and Rhodococcus spp. |
| Central-line associated blood stream infection [CLABSI]^69^ | For the purposes of CALIPSO study, this infection must occur within index acute healthcare admission after index cardiac surgery (excludes admission to rehabilitation or sub-acute care facilities). Time frame of measurement for this endpoint will be from date of index surgery until the date of discharge from the acute healthcare facility or date of death from any cause, whichever came first, assessed up to 30 days from index surgery.  AND  Was not found to be present or incubating at the time of index admission  AND  Must meet the criteria for BSI  AND  Must meet **at least one** the following criteria:   - 1. A BSI where central line was in place for >2 calendar days on the date of event, with day of device placement being Day 1   OR   - 1. A central line was in place on the date of event or the day before. If a central line was in place for >2 calendar days and then removed, the date of event of the BSI must be the day of discontinuation or the next day. If the patient is admitted or transferred into a facility with an implanted central line (port) in place, and that is the patient’s only central line, day of first access in an inpatient location is considered Day1. “Access” is defined as line placement, infusion or withdrawal through the line. Such lines continue to be eligible for CLABSI once they are accessed until they are either discontinued or the day after patient discharged. |
| Pneumonia [PNEU]^70,71^ | For the purposes of CALIPSO study, this infection must occur within index acute healthcare admission after index cardiac surgery (excludes admission to rehabilitation or sub-acute care facilities). Time frame of measurement for this endpoint will be from date of index surgery until the date of discharge from the acute healthcare facility or date of death from any cause, whichever came first, assessed up to 30 days from index surgery.  AND  Was not found to be present or incubating at the time of index admission  AND  Must meet **all** of the following criteria:   1. Chest imaging test result demonstrating    1. New and persistent radiological changes (infiltrate, consolidation, cavitation)   **OR**   - 1. Progressive and persistent radiological changes (infiltrate, consolidation, cavitation)   AND   1. At least **one** of the following criteria:    1. Fever (> 38.0°C)    2. Leukopenia (≤ 4000 WBC/mm3) or leucocytosis (≥ 12,000 WBC/mm3)    3. For adults ≥ 70 years old, altered mental status with no other recognized cause   AND  At least **one** of the following:   - - New onset of purulent sputum or change in character of sputum, or increased respiratory secretions, or increased suctioning requirement   - New onset or worsening cough, or dyspnoea, or tachypnoea (respiration rate > 25 breaths per minute)   - Rales (“crackle”) or bronchial breath sounds   - Worsening gas exchange (for example: O2 desaturations, increased oxygen requirements, or increased ventilator demand) |
| Infection-related Ventilator-Associated Complication [IVAC]^70,71^ | For the purposes of CALIPSO study, this infection must occur within index acute healthcare admission after index cardiac surgery (excludes admission to rehabilitation or sub-acute care facilities). Time frame of measurement for this endpoint will be from date of index surgery until the date of discharge from the acute healthcare facility or date of death from any cause, whichever came first, assessed up to 30 days from index surgery.  AND  Was not found to be present or incubating at the time of index admission  AND  Must meet the following criteria:   1. On or after calendar day 3 of mechanical ventilation and within 2 calendar days before or after the onset of worsening oxygenation   AND   1. **One** of the following criterion is met: 2. Criterion 1: Positive culture of **one of the following specimens**, meeting quantitative or semi-quantitative thresholds†, without requirement for purulent respiratory secretions:  - Endotracheal aspirate, ≥ 10^5^ CFU/ml or corresponding semi-quantitative result - Bronchoalveolar lavage, ≥ 10^4^ CFU/ml or corresponding semi-quantitative result - Lung tissue, ≥ 10^4^ CFU/g or corresponding semi-quantitative result - Protected specimen brush, ≥ 10^3^ CFU/ml or corresponding semi-quantitative result   OR   1. Criterion 2: Purulent respiratory secretions (defined as secretions from the lungs, bronchi, or trachea that contain ≥ 25 neutrophils and ≤ 10 squamous epithelial cells per low power field‡   AND  organism identified from one of the following specimens (to include qualitative culture, or quantitative/semi-quantitative culture without sufficient growth to meet Criterion #1):   - Sputum - Endotracheal aspirate - Bronchoalveolar lavage - Lung tissue - Protected specimen brush   OR   1. Criterion 3: **One of the following** positive tests:  - Organism identified from pleural fluid (where specimen was obtained during thoracentesis or within 24 hours of chest tube placement; pleural fluid specimens collected after a chest tube is repositioned or from a chest tube in place > 24 hours) - Lung histopathology, defined as abscess formation or foci of consolidation with intense neutrophil accumulation in bronchioles and alveoli - Diagnostic test for Legionella species   † If the laboratory reports semi-quantitative results, a semi-quantitative result of “moderate” “many” “numerous” or “heavy” growth, or 2+, 3+ or 4+ growth, meets the thresholds for Criterion 1.  ‡ If the laboratory reports semi-quantitative results a semi-quantitative result of “many”, “heavy”, “numerous,” or 4+, or ≥ 25 neutrophils per low power field meets the threshold for Criterion 2. |
| Urinary tract infection [UTI]^72^ | For the purposes of CALIPSO study, this infection must occur within index acute healthcare admission after index cardiac surgery (excludes admission to rehabilitation or sub-acute care facilities). Time frame of measurement for this endpoint will be from date of index surgery until the date of discharge from the acute healthcare facility or date of death from any cause, whichever came first, assessed up to 30 days from index surgery.  AND  Was not found to be present or incubating at the time of index admission  AND  Must meet **all** the following criteria:   - - - 1. One of the following is true:  1. Patient has/had an indwelling urinary catheter, but it has/had not been in place for more than two consecutive days on the date of event   OR   1. Patient did not have an indwelling urinary catheter in place on the date of event nor the day before the date of event   AND   1. Patient has at least one of the following signs or symptoms:  - fever (>38°C) - suprapubic tenderness* - costovertebral angle pain or tenderness* - urinary frequency - urinary urgency - dysuria  1. Patient has a urine culture with no more than two species of organisms identified, at least one of which is a bacterium of ≥10^5^ CFU/ml.   Comments:  * With no other recognized cause |
| Catheter-associated urinary tract infection [CAUTI]^72^ | For the purposes of CALIPSO study, this infection must occur within index acute healthcare admission after index cardiac surgery (excludes admission to rehabilitation or sub-acute care facilities). Time frame of measurement for this endpoint will be from date of index surgery until the date of discharge from the acute healthcare facility or date of death from any cause, whichever came first, assessed up to 30 days from index surgery.  AND  Was not found to be present or incubating at the time of index admission  AND  Must meet the following criteria:   - - - 1. Patient had an indwelling urinary catheter that had been in place for more than two consecutive days on the date of event AND was either  1. Present for any portion of the calendar day on the date of event   OR   1. Removed the day before the date of event   AND   1. Patient has at least one of the following signs or symptoms:  - fever (>38°C) - suprapubic tenderness* - costovertebral angle pain or tenderness* - urinary frequency - urinary urgency - dysuria  1. Patient has a urine culture with no more than two species of organisms identified, at least one of which is a bacterium of ≥10^5^ CFU/ml.   Comments:  * With no other recognized cause |

**3. Economic Endpoint Definitions**

| **ENDPOINT** | **DEFINITIONS** |
| --- | --- |
| Days alive and at home [DAH]^73^ | DAH will be calculated at Day 180 [DAH_180_] post index surgery. DAH will be calculated using mortality and hospitalisation data from the date of index surgery (=Day 0) until Day 180. DAH is calculated through subtracting the days from the index hospital length of stay, readmissions to hospital or admissions to rehabilitation or other nursing facility. If the participant dies within the 180 days, the DAH score is zero (0). |
| Direct health care costs | Cost data (in Australian dollars for Australian sites only) will be derived from hospital administrative databases and will include all services provided to individual patients assigned relevant service costs. Costs will only be collected for those participants meeting the primary trial endpoint (SSI) or secondary trial endpoint (HCAI). Services comprise specific, individualized items, such as medical imaging, pharmacy, pathology, and operation and the costs of consultation by medical, nursing, and allied health staff. Total inpatient costs associated with the index surgery will be collected, including inpatient rehabilitation costs, costs associated with attendance at outpatient clinics, as well as costs associated with any readmissions in the following 180 days, with the total combined costs defined herein as “episode of care” costs.^74^ |

# **4. Safety Endpoint Definitions**

| **ENDPOINT** | **DEFINITIONS** |
| --- | --- |
| Delayed antimicrobial hypersensitivity^26,75^ | For the purposes of CALIPSO study, this reaction must occur within index acute healthcare admission after index cardiac surgery (excludes admission to rehabilitation or sub-acute care facilities)  AND  Reaction occurs ≥24 hours after exposure to the study drug  Time frame of measurement for this endpoint will be from date of index surgery until the date of discharge from the acute healthcare facility or date of death from any cause, whichever came first, assessed up to 30 days from index surgery.  Comments:  The delayed hypersensitivity reaction will be further classified/categorised as   1. Mild – maculopapular rash (exanthum) without features of severe delayed hypersensitivity reaction 2. Severe including:    - Severe cutaneous adverse reactions (SCAR): cutaneous plus internal organ or mucous membrane involvement    - Drug rash with eosinophilia and systemic symptoms (DRESS): fever, eosinophilia, desquamative dermatitis and liver or kidney dysfunction    - Stevens–Johnson syndrome / toxic epidermal necrolysis (SJS/TEN): fulminant epidermal skin and epithelial mucosal loss    - Acute generalised exanthematous pustulosis (AGEP): multiple pin-sized pustules with erythema, fever and leucocytosis, and rarely, organ involvement    - Acute interstitial nephritis (AIN): acute kidney injury kidney +/- eosinophilia, fever and exanthematous rash.    - Serum sickness: vasculitic or urticarial rash, arthralgia/arthritis, fever, hypocomplementaemia and sometimes proteinuria.    - Drug-induced liver disease. |
| Immediate antimicrobial hypersensitivity^26,75^ | For the purposes of CALIPSO study, this reaction must occur within index acute healthcare admission after index cardiac surgery (excludes admission to rehabilitation or sub-acute care facilities)  AND  Reaction occurring <48 hours after exposure to the study drug.  Time frame of measurement for this endpoint will be from date of index surgery until the date of discharge from the acute healthcare facility or date of death from any cause, whichever came first, assessed up to 30 days from index surgery.  Comments:  The immediate hypersensitivity reaction will be further classified as   1. Mild - mild urticaria and/or rash 2. Severe - extensive urticarial rash, swelling of the face, extremities or throat (angioedema), wheezing or stridor, and/or hypotension collapse or anaphylaxis |
| All-cause mortality | Death reported up to 180 days from index cardiac surgery due to any cause. |
| SSI due to drug-resistant infections | In a participant meeting the primary endpoint for SSI, infection due to a drug-resistant infection will be defined as resistance to cefazolin, according to the European Committee on Antimicrobial Susceptibility Testing (EUCAST) -  2022, v 12.0 clinical breakpoints and dosing table for bacteria. |
| Acute kidney injury [AKI] | AKI will be defined according to the RIFLE criteria.^76^ For the purposes of CALIPSO study, this injury must occur within index acute healthcare admission after index cardiac surgery (excludes admission to rehabilitation or sub-acute care facilities).  AND  Must meet **at least one** of the following criteria:   1. Increase in serum creatinine to 1.5 times baseline   OR   1. Urine output of <0.5 mL/kg/hour for 6 to 12 hours   Time frame of measurement for this endpoint will be from date of index surgery until the date of discharge from the acute healthcare facility or date of death from any cause, whichever came first, assessed up to 30 days from index surgery.  Comments  The severity of AKI will be further graded as per the modified RIFLE criteria^76^*   1. Stage 0  - No increase or increase in serum creatinine <1.5 times baseline   OR   - Urine output of >0.5 mL/kg/hour  1. Stage 1 (Risk)  - Increase in serum creatinine to 1.5 times baseline   OR   - Urine output of <0.5 mL/kg/hour for 6 to 12 hours  1. Stage 2 (Injury)    - Increase in serum creatinine to 2 times baseline   OR   - - Urine output of <0.5 mL/kg/hour for 12 to 24 hours  1. Stage 3 (Failure)    - Increase in serum creatinine to 3 times baseline   OR   - - Increase in serum creatinine by >0.5 mg/dL to >4.0 mg/dL   OR   - - Urine output of <0.3 mL/kg/hour for >24 hours or anuria for >12 hours   OR   - - Initiation of kidney replacement therapy   * The criteria that lead to the worst possible classification should be used. |

**5. List of active sites (or sites with ethics/IRB approval that are awaiting activation) as of January 2026**

| **Site Name** | **Country** |
| --- | --- |
| The Alfred Hospital (Bayside Health), Melbourne, VIC | Australia |
| The Victorian Heart Hospital (Monash Health), Melbourne, VIC | Australia |
| The Royal Melbourne Hospital, Melbourne, VIC | Australia |
| The Prince Charles Hospital, Brisbane, QLD | Australia |
| St George Public Hospital, Sydney, NSW | Australia |
| St John of God Hospital Subiaco, Perth, WA | Australia |
| Westmead Hospital, Sydney, NSW | Australia |
| Royal Hobart Hospital, Hobart, TAS | Australia |
| Epworth Richmond, Melbourne, VIC, | Australia |
| Waikato Hospital, Hamilton, NI | New Zealand |
| Princess Alexandra Hospital, Brisbane, QLD | Australia |
| Cabrini Hospital, Melbourne, VIC | Australia |
| Flinders Medical Centre, Adelaide, SA | Australia |
| Flinders Private Hospital, Adelaide, SA | Australia |
| Royal Adelaide Hospital, Adelaide, SA | Australia |
| Christchurch Hospital, Christchurch, SI | New Zealand |
| Austin Health, Melbourne, VIC | Australia |
| Wellington Hospital, Wellington, NI | New Zealand |
| St Vincent’s Hospital, Melbourne, VIC | Australia |
| Sir Charles Gairdner Hospital, Perth, WA | Australia |
| Fiona Stanley Hospital, Perth, WA | Australia |
| Halifax Infirmary, Halifax, Nova Scotia | Canada |
| St Andrews War Memorial Hospital, Brisbane, QLD | Australia |
| Institut Jantung Negara Sdn Bhd, Kuala Lumpur | Malaysia |
| University of Texas Southwestern Medical Center, Dallas, Texas | USA |
