# Supplementary material for: Multicentre, adaptive, double-blind, three-arm, placebo-controlled, non-inferiority trial examining antimicrobial prophylaxis duration in cardiac surgery (CALIPSO): trial protocol
Source: BMJ Open. 2026 Mar 18;16(3):e115209. doi: 10.1136/bmjopen-2025-115209 (PMC13007059; doi:10.1136/bmjopen-2025-115209)
Supplement: Supplementary file 2 [file bmjopen-2025-115209.reviewer_comments.pdf]

# PEER REVIEW HISTORY

BMJ Open publishes all reviews undertaken for accepted manuscripts. Reviewers are asked to complete a checklist review form and are provided with free text boxes to elaborate on their assessment. These free text comments are reproduced below.

## ARTICLE DETAILS

**Title (Provisional)**

Multicentre, Adaptive, Double-Blind, Three-Arm, Placebo-Controlled, Noninferiority Trial Examining Antimicrobial Prophylaxis Duration In Cardiac Surgery (CALIPSO): Trial Protocol

**Authors**

Peel, T.; McGiffin, David; Smith, Julian; Forbes, Andrew; Marasco, Silvana; Pilcher, David; Stewardson, Andrew; Petrie, Dennis; Peleg, Anton; Wisniewski, Jessica; Forster, Samuel; Druce, Paige Elizabeth; Roney, Janine; Astbury, Sarah; Berkovic, Danielle; Mccracken, Phoebe; Steering Committee, CALIPSO Trial; Myles, PS

## VERSION 1 - REVIEW

| | |
|---|---|
| **Reviewer** | **1** |
| **Name** | **Boisson, Matthieu** |
| **Affiliation** | **Centre Hospitalier Universitaire de Poitiers, Anaesthesia and Intensive Care Unit** |
| **Date** | **13-Jan-2026** |
| **COI** | **None** |

This is a very important, timely and ambitious protocol addressing a long-standing and clinically relevant question in cardiac surgery, namely the optimal duration of antimicrobial prophylaxis after sternotomy. The CALIPSO trial is clearly large-scale, methodologically sophisticated, and addresses an important gap in a field where practice remains heterogeneous despite international guidelines.

Overall, the trial design is robust, the adaptive multi-arm approach is well justified, the blinding and endpoint adjudication procedures are of high quality, and the integration of clinical, safety and health economic outcomes is a major strength. The protocol is clearly suitable for publication in BMJ Open. My comments below are not intended to request major changes to the study design, but rather to seek clarifications and strengthen the scientific and methodological transparency of the manuscript.

Major comments

1. Assumed baseline SSI rate for sample size calculation

The sample size and simulations are based on an assumed baseline SSI incidence of 8%. This figure appears relatively high compared with several contemporary cardiac surgery cohorts and recent trials using modern prevention bundles and surveillance definitions (often reporting rates closer to 2–5%, depending on case-mix and definitions).

While this assumption cannot be changed at this stage, it would be important for the authors to clarify:

• On which contemporary data (local, national, or multicentre surveillance) this 8% estimate is based.

• How a substantially lower observed SSI rate would affect the interpretation of the non-inferiority margin (2.5%) in clinical terms, even if the statistical framework remains valid.

A short discussion of this point would help readers to better contextualise the trial results when they become available.

2. Description and justification of the prophylaxis regimenDescription and justification of the prophylaxis regimen (including intraoperative strategy)

The postoperative regimen consists of fixed-dose cefazolin 2 g every 8 hours. In contemporary cardiac surgery populations, which frequently include obese patients and patients with major physiological derangements after cardiopulmonary bypass, this dosing strategy may not always ensure optimal or homogeneous exposure.

It would be useful for the authors to:

• Provide a clearer justification for the choice of this dosing regimen in the postoperative period.

• Acknowledge and discuss the possibility that prolonged prophylaxis arms may not represent a pharmacologically “optimised” strategy, but rather a pragmatic extension of current common practice.

This does not undermine the trial, but it is important for the interpretation of both efficacy and safety results.

In addition, the intraoperative prophylaxis strategy itself is only described in broad terms (e.g., administration within 60 minutes before incision and re-dosing for prolonged procedures according to local practice). Given the central role of cardiopulmonary bypass in cardiac surgery and its known impact on antibiotic pharmacokinetics, it would be important to clarify:

Whether a systematic re-dosing at the time of initiation of cardiopulmonary bypass is recommended, required, or merely left to local practice.

How intraoperative re-dosing (timing, triggers such as duration of surgery or blood loss) will be documented.

Whether these intraoperative practices will be monitored and reported, at least descriptively.

Although the trial focuses on postoperative prophylaxis, variability in intraoperative antibiotic exposure could meaningfully influence the primary outcome and its interpretation. A clearer description of how intraoperative prophylaxis is handled and captured in the study would therefore be very helpful.

4. Co-interventions and other SSI prevention measures

The pragmatic nature of the trial is appropriate and clearly justified. However, many other perioperative measures are known to influence SSI risk in cardiac surgery (e.g., skin decolonisation, antisepsis protocols, glycaemic control, temperature management, intraoperative re-dosing strategies, especially around cardiopulmonary bypass).

At present, it is not entirely clear which of these co-interventions will be:

• Systematically recorded,

• And whether they will be reported descriptively or used in any secondary or sensitivity analyses.

Even in a pragmatic trial, documenting key co-interventions is important to interpret the results and to assess balance between arms. Clarifying this point in the protocol would strengthen the methodological transparency.

5. Post-discharge follow-up procedures

The protocol states that patients will be followed up to 180 days and that outcomes will be assessed at days 30, 90 and 180, but the practical modalities of follow-up after hospital discharge are not described in much detail.

It would be helpful to specify:

• How follow-up will be conducted (telephone calls, clinic visits, electronic health records, contact with primary care, etc.).

• How suspected events outside the index hospital will be captured.

• What procedures are in place to minimise and manage loss to follow-up.

Given that the primary endpoint is assessed at 90 days, this information is important to judge the robustness of outcome ascertainment.

Minor comments

• The manuscript might benefit from a brief clarification of how the estimand framework (treatment policy vs hypothetical estimand) aligns with current ICH E9(R1) guidance, although the planned analyses themselves are appropriate and clearly described.

• Consider explicitly stating whether superficial, deep and organ/space SSIs will be reported separately as secondary or exploratory analyses, in addition to the composite primary endpoint.

• As this is an ongoing trial with recruitment already started, it would be helpful to report the number of patients already enrolled at the time of submission, to provide readers with a clearer view of the study's current status.

Conclusion

This is a high-quality, well-designed and much-needed trial that addresses an important and unresolved clinical question. The above comments are intended to improve clarity, transparency and interpretability of the protocol rather than to request any fundamental changes to the study. I strongly support publication of this protocol in BMJ Open after these points are addressed.

---

## VERSION 1 - AUTHOR RESPONSE

| | Reviewer(s) Comment | Authors' Response | Manuscript location (tracked version) |
|---|---|---|---|
| **Major comments** | | | |
| **1A.** | Assumed baseline SSI rate for sample size calculation<br>The sample size and simulations are based on an assumed baseline SSI incidence of 8%. This figure appears relatively high compared with several contemporary cardiac surgery cohorts and recent trials using modern prevention bundles and surveillance definitions (often reporting rates closer to 2–5%, depending on case-mix and definitions).<br>While this assumption cannot be changed at this stage, it would be important for the authors to clarify: On which contemporary data (local, national, or multicentre surveillance) this 8% estimate is based. | The baseline SSI of 8% was obtained from the incidence of SSI from a post-hoc analysis of the ATACAS trial (Myles. N Engl J Med 2016. 10.1056/NEJMoa1606424). The post hoc analysis looked at a subset of 613 patients from our institution (Alfred Hospital) that were enrolled in the ATACAS Trial. The incidence SSI was of 17.5% (107/613), including 92 superficial, 17 deep and 5 organ/space infections (Draxler. Blood Adv 2019. 10.1182/bloodadvances.2019000092). We opted for a more conservative estimate of 8% which was in keeping with the incidence published by Tamayo et al (Tamayo. J Thorac Cardiovasc Surg 2008. 10.1016/j.jtcvs.2008.05.013).<br><br>Of note, the Victoria SSI Surveillance reports (all hospitals undertaking cardiac surgery must report their results to VICNISS including a number of the hospitals recruiting to our trial) report an incidence of 4.92 (4.64–5.22) without robust post-operative follow up (Worth. Inf Contr Hosp Epi. 2015. 10.1017/ice.2014.70).<br><br>This is similar to many other publications that do not include robust post-discharge SSI surveillance, relying on representation to hospital to capture the outcome. Therefore, these studies often miss infections (particularly superficial infections). | Page 5, lines 39-42. |

| | | | |
|---|---|---|---|
| 1B | How a substantially lower observed SSI rate would affect the interpretation of the non-inferiority margin (2.5%) in clinical terms, even if the statistical framework remains valid. | In terms of statistical power, for a fixed absolute NI margin of 2.5%, the power is actually greater the lower the SSI rate. That is because the random variation (i.e. variance) around a proportion P is P*(1-P)/N, which is greatest when P=50%, and reduces as P gets smaller. So precision (and hence power) with SSI rate of P=5% is greater than with P=8%.<br><br>For example, the protocol paper states:<br>When the true SSI rates are 8% in each arm, the probability of correctly declaring noninferiority (power) of the intraoperative arm compared to either of 24- and 48-hour arms is 88%, and power for simultaneous noninferiority compared to both these arms is 80%;<br><br>Redoing the numerical simulations replacing 8% by 5%, the power is greater:<br>When the true SSI rates are **5%** in each arm, the probability of correctly declaring noninferiority (power) of the intraoperative arm compared to either of 24- and 48-hour arms is **98%**, and power for simultaneous noninferiority compared to both these arms is **96%**; | |
| 2. | The postoperative regimen consists of fixed-dose cefazolin 2 g every 8 hours. In contemporary cardiac surgery populations, which frequently include obese patients and patients with major physiological derangements after cardiopulmonary bypass, this dosing strategy may not always ensure optimal or homogeneous exposure.<br>It would be useful for the authors to:<br>• Provide a clearer justification for the choice of this dosing regimen in the postoperative period.<br>• Acknowledge and discuss the possibility that prolonged prophylaxis arms may not represent a pharmacologically "optimised" strategy, but rather a pragmatic extension of current common practice.<br>This does not undermine the trial, but it is important for the interpretation of both efficacy and safety results. | The fixed dose was based on the Australian Therapeutic Guideline recommendations (2019) which is the national guideline and was a pragmatic extension of current common practice.<br><br>Much of the data on "pharmacologically optimised strategies" are from treatment studies (rather than prophylaxis studies) therefore may differ for prophylaxis approaches. That said, we acknowledge that the selected dosing approach may not achieve homogeneous exposure. | Page 7, lines 41-43. |
| 3. | The intraoperative prophylaxis strategy itself is only described in broad terms (e.g., administration within 60 minutes before incision and re-dosing for prolonged procedures according to local | Given the pragmatic nature of the trial and the fact that the focus is on postoperative dosing, we have not been overly proscriptive for intraoperative dosing and allow sites to dose according to national guidelines (like the Therapeutic Guidelines in Australia) or local | Page 8, lines 23-27. |

| | | | |
|---|---|---|---|
| | practice). Given the central role of cardiopulmonary bypass in cardiac surgery and its known impact on antibiotic pharmacokinetics, it would be important to clarify:<br>Whether a systematic re-dosing at the time of initiation of cardiopulmonary bypass is recommended, required, or merely left to local practice.<br>How intraoperative re-dosing (timing, triggers such as duration of surgery or blood loss) will be documented.<br>Whether these intraoperative practices will be monitored and reported, at least descriptively. | policy. In the Therapeutic Guidelines, re-dosing is recommended if the procedure extends beyond 3-4 hours or if there is substantial blood loss (~1.5L) – there is no specific recommendation regarding timing with bypass.<br><br>We are collecting information on all aspects of intraoperative antimicrobial prophylaxis including re-dosing (including the time, dose and method of administration noting that some centres administer the antibiotic into the bypass pump), as well as operation time and commencement and cessation (and duration) of bypass. These factors will be reported and will be incorporated into the estimand framework. | |
| **4.** | Co-interventions and other SSI prevention measures<br>The pragmatic nature of the trial is appropriate and clearly justified. However, many other perioperative measures are known to influence SSI risk in cardiac surgery (e.g., skin decolonisation, antisepsis protocols, glycaemic control, temperature management, intraoperative re-dosing strategies, especially around cardiopulmonary bypass).<br>At present, it is not entirely clear which of these co-interventions will be:<br>• Systematically recorded,<br>• And whether they will be reported descriptively or used in any secondary or sensitivity analyses. | We are systematically collecting information around other infection prevention approaches, including screening and decolonisation and surgical site skin preparation etc and operative factors (duration, bypass etc). They will be reported descriptively. Given the large (N=9180) trial design, we anticipate very good balance of these covariates across the 3 groups. | Page 8, lines 27-29. |
| **5.** | Post-discharge follow-up procedures<br>The protocol states that patients will be followed up to 180 days and that outcomes will be assessed at days 30, 90 and 180, but the practical modalities of follow-up after hospital discharge are not described in much detail.<br>It would be helpful to specify:<br>• How follow-up will be conducted (telephone calls, clinic visits, electronic health records, contact with primary care, etc.).<br>• How suspected events outside the index hospital will be captured.<br>• What procedures are in place to | The processes for identifying outcomes will be conducted by the project research officer who will collect demographic data and relevant laboratory testing, operative data and quality of life (EQ-5D-5L). Active surveillance will comprise the following:<br>● Review of all participants' medical records on discharge and follow-up<br>● Telephone contact for outcomes and quality of life data at 30-, 90- & 180-days following index surgery<br>● Contact with the participant's GP and other healthcare institutions as required,<br>● Clinical microbiological reports with antimicrobial susceptibility data for SSI and HCAI isolates | Page 8, lines 46-50; Page 9 lines 1-4. |

| | | | |
|---|---|---|---|
| | minimise and manage loss to follow-up. | We have applied these same processes for our other clinical trials (eg. Peel NEJM 2023, Corcoran NEJM 2021) with <2% loss to follow up. | |
| Minor Comments | | | |
| | The manuscript might benefit from a brief clarification of how the estimand framework (treatment policy vs hypothetical estimand) aligns with current ICH E9(R1) guidance, although the planned analyses themselves are appropriate and clearly described. | Compliance with ICH E9(R1) explicitly stated. | Page 10, lines 19-20. |
| | Consider explicitly stating whether superficial, deep and organ/space SSIs will be reported separately as secondary or exploratory analyses, in addition to the composite primary endpoint. | Superficial, deep and organ/space SSIs will be reported separately as secondary or exploratory analyses, in addition to the composite primary endpoint. | Page 9, lines 19-20 |
| | As this is an ongoing trial with recruitment already started, it would be helpful to report the number of patients already enrolled at the time of submission, to provide readers with a clearer view of the study's current status. | Total recruitment to date (as of 12:00PM AEST Thursday 29th of January, 2026) has been included. | Page 4. Line 28. |
